# Predictors of Extubation Failure in Neurocritical Patients Identified by a Systematic Review and Meta-Analysis

**DOI:** 10.1371/journal.pone.0112198

**Published:** 2014-12-08

**Authors:** Shengnan Wang, Lili Zhang, Kaibin Huang, Zhenzhou Lin, Weiguang Qiao, Suyue Pan

**Affiliations:** 1 Department of Neurology, Nanfang Hospital, Southern Medical University, Guangzhou 510515, China; 2 Department of Neurology, The second people's hospital of chengdu, Chengdu 610017, China; 3 Department of Gastroenterology, Nanfang Hospital, Southern Medical University, Guangzhou 510515, China; Weill Cornell Medical College, United States of America

## Abstract

**Background:**

Prediction of extubation failure, particularly in neurocritical patients, is unique and controversial. We conducted a systematic review and meta-analysis to identify the risk factors for extubation failure in these patients.

**Methods:**

A literature search of databases (MEDLINE, EMBASE, the Cochrane Library, and Web of Science) was performed up to August of 2013 to identify trials that evaluated extubation failure predictors. Included trials were either prospective or retrospective cohort studies.

**Results:**

Nine studies involving 928 participants were included. The systematic review and meta-analysis revealed that the following were predictive for extubation failure: pneumonia, atelectasis, mechanical ventilation of >24 h, a low Glasgow Coma Scale score (7–9T) (OR = 4.96, 95% CI = 1.61–15.26, P = 0.005), the inability to follow commands (OR = 2.07, 95% CI = 1.15–3.71, P = 0.02), especially the command to close the eyes, thick secretion, and no intact gag reflex. Meanwhile, the following were not predictive for extubation failure: sex, secretion volume, coughing upon suctioning, and the inability to follow one command among showing two fingers, wiggling the toes, or coughing on command. Additionally, some traditional weaning parameters were shown to poorly predict extubation failure in neurocritical patients.

**Conclusions:**

Besides pneumonia, atelectasis, and the duration of mechanical ventilation, other factors that should be taken into consideration in the prediction of extubation failure when neurocritical patients are weaned from tracheal intubation include neurologic abilities (Glasgow Coma Scale score and following commands), the secretion texture, and the presence of a gag reflex.

## Introduction

Many patients in the neurocritical care unit (NCU) require intubation for airway management. Both delayed and premature extubation increase the rates of complications, need for tracheotomy, duration of intensive care unit (ICU) stay, and mortality [1]–[4]. Thus, it is crucial to predict whether and when to extubate such patients.

Many clinical trials have focused on the predictors of extubation failure (EF) to refine clinical and laboratory indices in the assessment of extubation readiness [1], [2]. One review summarized the predictors of EF in the general ICU as follows: demographic predictors, predictors assessing respiratory mechanics, parameters assessing airway protection, parameters assessing airway patency, and parameters assessing hemodynamics and tissue perfusion [5]. However, the risk factors for EF vary extensively with the different disease states among patients.

Prediction of EF among patients in the NCU is even more controversial, and may be related to specific factors [3], such as the inability to protect the airway because of disturbance of consciousness or bulbar palsy even after traditional weaning parameters (TWPs) have been met; requiring amounts of fluid infusion to ensure cerebral perfusion; respiratory failure caused by respiratory center dysfunction and peripheral neuropathy or neuromuscular disease, but not just pulmonary pathology [6]. Furthermore, Ko [6] demonstrated neither individual TWPs nor combinations of TWPs that can be used for EF prediction in neurocritical patients.

Because of the specific management requirements for patients in the NCU, most studies have focused on the level of consciousness [including the Glasgow Coma Scale (GCS) score and Full Outline of Unresponsiveness (FOUR) score] and the ability to ensure airway protection [3], [7]–[10]. Although no association between the GCS score and EF was identified in one prospective study [3], a higher GCS score is still preferred for extubation [7], [10]–[12]. Neurologic impairment was found to be another independent risk factor for EF in other studies [9], [10], [13]. Moreover, Salam [8] and Anderson [7] demonstrated that the ability to follow four commands (open eyes, follow with eyes, grasp hand, and stick out tongue vs. close eyes, show two fingers, wiggle toes, and cough on command) was a significant predictor of successful extubation.

These discrepancies might be due to the insufficient statistical power of individual studies and the inability to perform separate analyses. Therefore, we conducted this systematic review and meta-analysis to identify the risk factors for EF in neurocritical patients.

## Materials and Methods

### Literature and search strategy

A comprehensive literature search was carried out. The literature search was updated to August 2013 and included multiple databases (MEDLINE, EMBASE, the Cochrane Library, and Web of Science). Some relevant articles were searched manually. The search strategy to identify all possible studies involved the use of the following keywords: “extubation” and (“factor*” or “predict*”) and (“neuro*” or “brain-injured” or “cerebral”). The search was restricted to English-language papers. When the data were not readily extractable, standard letters seeking to clarify the original datasets were sent to the corresponding authors.

### Inclusion and exclusion criteria

Two authors independently identified and screened the search results for potentially eligible studies. Inclusion or exclusion of articles was determined by two independent investigators (S.N.W. and L.L.Z.). Discrepancies were discussed and resolved by agreement or consultation with a third author (S.Y.P.).

The inclusion criteria were as follows: (1) original research on predicting extubation in NCU patients >18 years of age, (2) English-language studies with full texts, (3) human studies, and (4) studies with available data. For articles that shared the same data, the article with the most information was selected.

The exclusion criteria were as follows: (1) abstracts, letters, editorials, expert opinions, reviews, and case reports; (2) articles without sufficient data for calculation of odds ratios (ORs) or relative risk (RR) with 95% confidence intervals (CIs); (3) low-quality studies; (4) studies that did not assess extubation predictors specially for NCU patients; and (5) studies of tracheostomy tube decannulation or anesthesia.

### Data extraction and quality assessment

Two investigators (S.N.W. and L.L.Z.) independently extracted and tabulated the data from each study, including the author, country, design, period of data collection, year of publication, participants (sample size, inclusion and exclusion criteria), definition of EF, incidence of EF, and risk factors related to EF.

Study quality was independently assessed by two investigators (S.N.W. and L.L.Z.) using the Newcastle–Ottawa Scale (NOS) [14], which specifically considers the selection of the case, comparability between the exposed and nonexposed group, and outcome assessment and follow-up. Studies with a NOS score of ≧5 were considered to be high-quality studies and were included in this systemic review and meta-analysis.

### Statistical analysis

Statistical analyses were conducted with Review Manager (version 5.2; The Cochrane Collaboration). A Q-test was performed to examine between-study heterogeneity. The pooled outcomes were assessed by OR and 95%CI. A random- or fixed-effects model was used to calculate the pooled OR in the presence (P<0.10) or absence (P>0.10) of heterogeneity, respectively. The I^2^ statistic was used to measure the percentage of total variation across the studies due to heterogeneity (I^2^ of ≧50% indicated the presence of heterogeneity). The significance of the pooled OR was determined by the Z test (P<0.05 was considered statistically significant). We performed sensitivity analyses to assess the stability of the results by evaluating whether the statistical model (fixed-effects vs. random-effects model) would change the results and then determine whether the publication quality (low or high quality) could influence the results of our systemic review and meta-analysis. Publication bias was quantitatively assessed by Begg's test [15] and Egger's test [16] (P<0.05 was considered statistically significant) using STATA (Version 12.0; StataCorp LP). Funnel plots were created to qualitatively demonstrate publication bias in the studies. A symmetrical funnel plot indicated effective control of publication bias.

## Results

### Results of literature search

**Figure 1:**
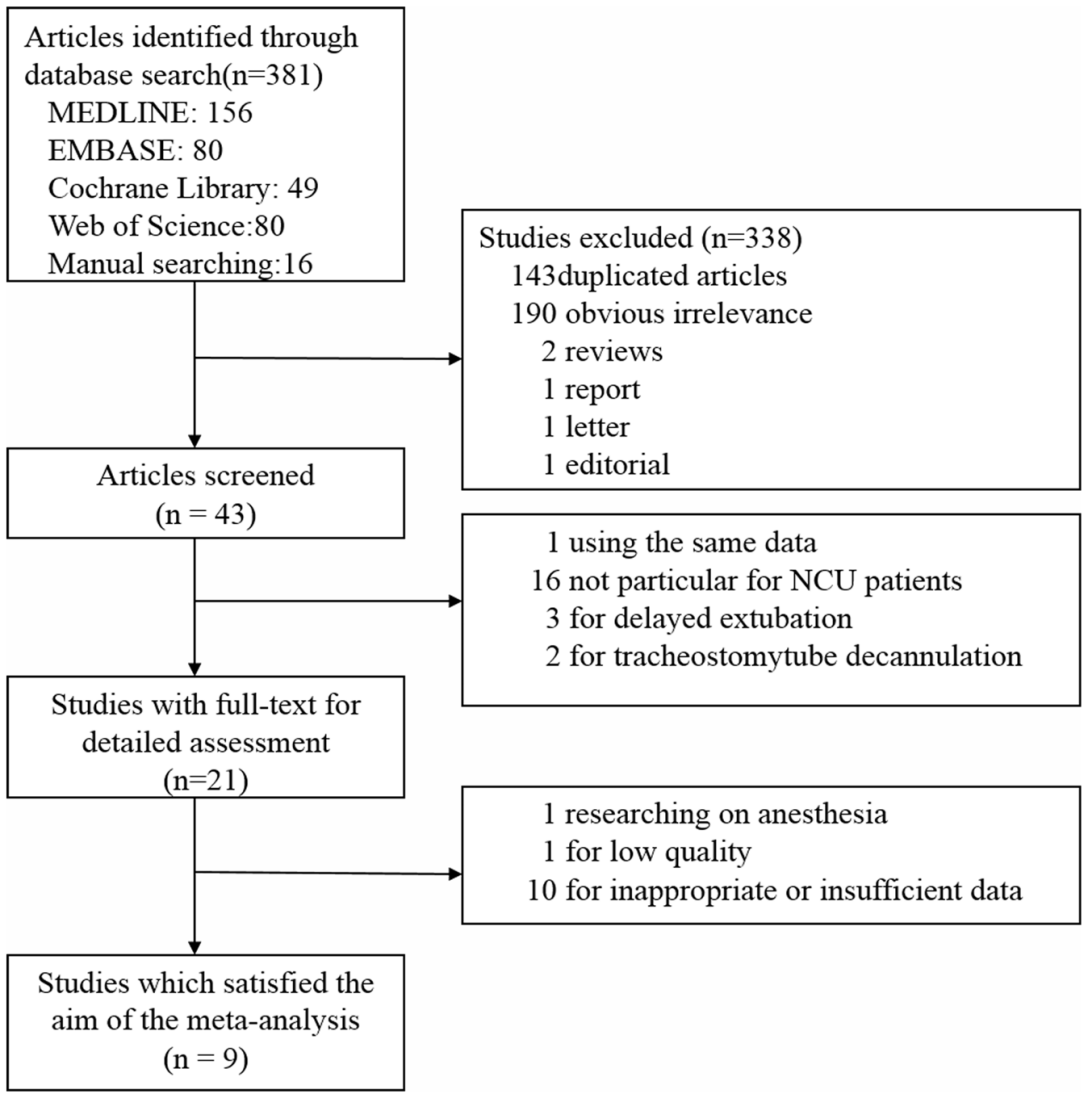
Flow chart illustrating the process of identifying relevant studies.

Details of the literature search are shown in Fig. 1. In total, 43 clinical studies were identified after screening. Thirty-four studies were excluded: 16 did not specifically study NCU patients, 1 used the same data as another that was included in the systematic review, 3 studied delayed extubation, and 2 focused on tracheostomy tube decannulation. Another 10 articles was excluded because of inappropriate or insufficient data, 1 paper researched on anesthesia and 1 paper was excluded for low quality. Finally, nine studies were included in the systemic review and meta-analysis.

### Description of the included papers

Table 1 presents the details of the included studies and the patients' characteristics. With respect to the study design, four trials were prospective cohort studies and five were retrospective cohort clinical trials. The reported incidence rates of EF for neurocritical patients ranged from 8.2% to 43.5%. The lowest and highest samples comprised 20 and 317 patients, respectively. The NOS score of all studies ranged from 5 to 8 (Table 2).

**Table 1:** Characteristics of included studies.

Author, Year, Country, Period of Data Collection	Design	Participants	Definition of failed extubation	Failed extubation incidence	Factors found in articles
1. Richard Ko, 2009, USA, during an 8 month period of examination [6]	A retrospective case series study	N = 62. Included: patients intubated due to cranial pathology. Excluded: patients with intubation due to spinal cord pathology, medical reasons, performance of a procedure, care withdrawn or brain dead, and missing data.	Need for reintubation within 48 h.	11/62(17.7%)	None
2. Christopher D.Anderson,2011,USA, May 2007 through December 2009 [7]	A prospective cohort study	N = 285. Included: patients with the primary diagnosis of (CNS) or (PNS) disease, suitable for extubation or tracheostomy, intubation duration of >6 h, GCS>6T, RSBI<105.Excluded: patients extubated in a terminal setting.	Require reintubation within 72 h. Individuals reintubated for a planned procedure were not counted as failures.	48/285(16.8%)	The ability to follow four commands(close eyes, show two fingers, wiggle toes, cough to command)
3.LindaC.Wendell,2011, USA, between January 2004 and December 2008 [9]	A retrospective cohort study	N = 47. Included: patients with Middle Cerebral Artery Acute Ischemic Stroke, onset of stroke symptoms within 24 hours of admission or transfer. Excluded: patients with direct tracheostomy without extubation attempt, extubation terminally, or being dead prior to an extubation attempt.	Requiring reintubation within 48 hours.	10/47(21.3%)	Extubation composite and eye response Glasgow Coma Scale (GCS) scores
4. Milena C. Vidotto, 2012, Brazil, from July 2005 to July 2009 [13]	A prospective observational cohort study	N = 317. Included: patients in the postoperative period of non-emergency intracranial surgery. Excluded: patients who died, underwent tracheostomy prior to weaning.	Requiring reintubation within 48 hours.	26/317(8.2%)	Lower level of consciousness (GCS 8T-10T) and female sex
5. Milena C. Vidotto, 2008,Brazil,Between July 2002 and July 2006 [17]	A prospective cohort study	N = 92. Included: patients required mechanical ventilation for up to 6 h after elective craniotomy. Excluded: patients who underwent tracheostomy, unplanned extubation or died before weaning trial, were intubated before surgery.	Need for reintubation within 48 h.	15/92(16.3%)	None
6.Jenn-Yu Wu, 2009, Taiwan, from January 2000 to June 2007 [18]	A retrospective cohort study	N = 33. Included: patients intubated for mechanical ventilation or supported by NIV. Excluded: patients with intubation for elective surgery, out hospital cardiac arrest, a duration of mechanical ventilation<48 h, and tracheostomy before admission were excluded.	Inability to spontaneous breathing or received NIV support within 72 h.	13/33(39.4%)	A maximal expiratory pressure (Pemax) of ≧40 cmH2O
7. Antonio A. M. Castro, 2012, Brazil, between July and October 2008 [19]	A prospective cohort study	N = 20.Included: patients with stroke (brain stem infarction), intubated (≧10 days), hemodynamically stable, (GCS) ≧7, infection controlled. Excluded: patients who presented arrhythmia, high blood pressure (MAP >150 mmHg) or low blood pressure (PAM<60 mmHg), recurring stroke of any type or any other disease other than the stroke.	Requiring reintubation within 48 hours.	8/20(40%)	A high RSBI, Pdi and low PaO_2_/FiO_2_ Pdi/Pdimax, Raw
8.Janaka Seneviratne, 2008,America, from January 1,1987,through December 31, 2006 [20]	A retrospective cohort study	N = Forty patients with 46 episodes of MC. Included: patients had severe generalized and bulbar weakness and were intubated for neuromuscular respiratory failure. Excluded: patients with postthymectomy, Lambert-Eaton syndrome, congenital myasthenia, and intubated for cardiac failure or pulmonary disease.	Reintubation (occurred within the same ICU admission and within 72 hours of extubation), tracheostomy, or death while intubated.	20/46(43.5%)	Male sex, history of previous crisis, atelectasis, and intubation for more than 10 days
9. Alejandro A. Rabinstein, 2005, USA, between 1996 and 2003 [21]	A retrospective cohort study	N = 26 episodes of MC in 20 patients. Included: patients requirement of mechanical ventilation due to neuromuscular respiratory failure. Excluded: patients with postthymectomy crises, tracheostomy.	Need for reintubation within 72 hours from extubation due to persistent respiratory insufficiency.	7/26(26.9%)	Older age, atelectasis, pneumonia

**Table 2:** The quality assessment of individual studies included.

Study included	Selection	Comparability	Outcome	Total
1. Richard Ko [6]	3	2	2	7
2.Christopher D. Anderson [7]	4	2	2	8
3.Linda C.Wendell [9]	3	2	1	6
4.Milena C.Vidotto [13]	4	2	2	8
5. Milena C. Vidotto [17]	4	2	1	7
6.Jenn-Yu Wu [18]	3	2	1	6
7. Antonio A. M. Castro [19]	4	2	1	7
8.Janaka Seneviratne [20]	3	2	1	6
9. Alejandro A. Rabinstein [21]	2	2	1	5

### Meta-analysis results

#### Demographic predictors of EF

A meta-analysis of seven studies [7], [9], [13], [17]–[20] showed that sex was not associated with extubation outcomes in NCU patients. Meanwhile, pneumonia [6], [7], [9], [17], [18], [20], [21], atelectasis [18], [20], [21], and a >24-h duration of mechanical ventilation (MV) [13], [17] were associated with EF in neurocritical patients. Sensitivity analyses performed by changing the statistical models showed stable results (Table 3). Publication bias was not present according to Begg's test and Egger's test. Considering there might exist any differences between the risk factors of EF in neuromuscular disease patients and intracranial disease patients, we excluded the researches focus on neuromuscular disease [20], [21], then re-analyzed the rest of studies [6], [7], [9], [17], [18]. The results showed that pneumonia was still associated with EF. Notwithstanding, meta-analysis of neuromuscular disease patients [20], [21] in a separate way found that atelectasis was associated with EF (Table 3).

**Table 3:** Results of meta-analysis in predicting extubation failure in neurocritical patients.

Comparison	Trials(n)	Participants (n)	Sensitivity analysis		P value^a^	Heterogeneity^b^
			Random-effect	Fixed-effect		
male vs. female	7	840	0.97[0.51, 1.86]	0.85[0.58, 1.26]	0.93	I^2^ = 50%, p = 0.06
pneumonia vs. non-pneumonia						
with MC^c^	7	593	4.19[1.57, 11.14]	2.74[1.80, 4.15]	0.004	I^2^ = 72%, p = 0.002
without MC	5	521	4.23 [1.18, 15.18]	2.60 [1.64, 4.12]	0.03	I^2^ = 79%, p = 0.0009
atelectasis vs. non-atelectasis						
total	3	105	5.96 [1.69, 21.03]	6.64 [2.44, 18.06]	0.0002	I^2^ = 21%, p = 0.28
MC only	2	72	9.46 [2.78, 32.18]	10.37 [3.11, 34.59]	0.0003	I^2^ = 0%, p = 0.50
MV^d^>24 h vs. MV≤24 h	2	409	2.63 [1.34, 5.15]	2.60 [1.32, 5.13]	0.006	I^2^ = 0%, p = 0.73
RSBI^e^>105 vs. RSBI≤105	3	187	2.40 [0.82, 7.06]	2.36 [0.81, 6.87]	0.12	I^2^ = 0%, p = 0.78
MV^f^≤10 l/min vs. MV>10 l/min	2	154	1.59 [0.41, 6.25]	1.65 [0.71, 3.83]	0.51	I^2^ = 59%, p = 0.12
PaO_2_/FiO_2_≤300vs. PaO_2_/FiO_2_>300	2	347	1.24 [0.69, 2.22]	1.24 [0.69, 2.22]	0.48	I^2^ = 0%, p = 0.97
TV^g^<5 mg/kg vs. TV≧5 mg/kg	2	95	1.41 [0.52, 3.84]	1.41 [0.52, 3.84]	0.50	I^2^ = 0%, p = 0.95
GCS^h^7-9T vs. GCS10-11T	3	693	4.96 [1.61,15.26]	3.48 [1.92, 6.33]	0.005	I^2^ = 65%, p = 0.06
Not following commands vs. following command	2	332	2.06 [1.14, 3.73]	2.07 [1.15, 3.71]	0.02	I^2^ = 0%, p = 0.34

a p value of effect size; ^b^p value for heterogeneity; ^c^ myasthenic crisis; ^d^duration of mechanical ventilation; ^e^rapid shallow breathing index; ^f^minute ventilation; ^g^tidal volume; ^h^Glasgow Coma Scale.

#### Predictors assessing respiratory mechanics

The following studies underwent a meta-analysis and showed that none of the following factors were associated with EF in neurocritical patients: three studies [6], [17], [18], rapid shallow breathing index of >105 vs. ≤105 (OR  =  2.36, 95% CI = 0.81–6.87, P = 0.12); two studies [6], [17], minute ventilation of ≤10/min vs. >10/min (OR = 1.59, 95% CI = 0.41–6.25, P = 0.51); two studies [6], [7], PaO_2_/FiO_2_ of ≤300 vs. >300 (OR = 1.24, 95% CI = 0.69–2.22, P = 0.48); and two studies [6], [18], tidal volume of <5 vs. ≧5 mg/kg (OR = 1.41, 95% CI = 0.52–3.84, P = 0.50). Neither a random-effects model nor a fixed-effects model changed the results (Table 3). Funnel plots revealed no asymmetry, indicating that there was no publication bias. However, the funnel plot analysis was limited in some cases because of the low number of studies.

#### Neurological status affecting extubation outcomes

**Figure 2:**
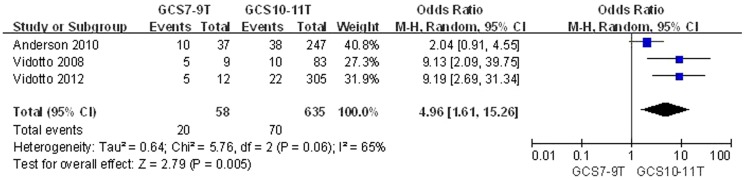
Meta-analysis result of GCS.

Data comparing a GCS score of 7–9T with a GCS score of 10–11T at the time of extubation among three included studies [7], [13], [17] revealed a significant difference in the increased risk of EF between the two groups (OR = 4.96, 95% CI = 1.61–15.26, P = 0.005). The reliability of this result was confirmed by the same result from the sensitivity analyses using a fixed-effects model (OR = 3.48, 95% CI = 1.92–6.33, P<0.0001) (Fig. 2, Table 3). No publication bias was identified (Begg's test, P = 1.000; Egger's test, P = 0.189).

**Figure 3:**
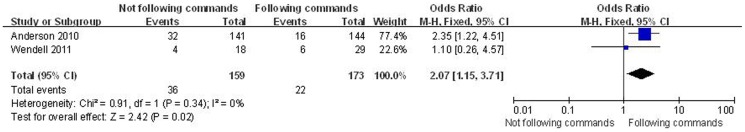
Meta-analysis result of following commands.

Two studies [7], [9] reported that the inability to follow commands (close eyes, show two fingers, wiggle toes, or cough on command vs. defined by a GCS motor score of 6) increased the risk of EF (OR = 2.07, 95% CI = 1.15–3.71, P = 0.02) (Fig. 3, Table 3).

Anderson [7] found that the inability to follow the command to close the eyes (OR = 2.06, 95% CI = 1.08–3.92, P = 0.03) increased the probability of EF. The inability to follow the command to show two fingers (OR = 1.57, 95% CI = 0.83–2.97, P = 0.16), wiggle the toes (OR = 1.41, 95% CI = 0.72–2.75, P = 0.31), and cough on command (OR = 1.10, 95% CI = 0.23–5.27, P = 0.90) did not predict EF.

#### Predictors assessing airway protection

Meanwhile, Anderson [7] found that a thick secretion texture (OR = 2.23, 95% CI = 1.11–4.49, P = 0.02) and no intact gag reflex (OR = 2.38, 95% CI = 1.21–4.68, P = 0.01) predicted EF. Neither the secretion volume (OR = 0.49, 95% CI = 0.06–3.93, P = 0.50) nor coughing with suctioning (OR = 1.10, 95% CI = 0.23–5.27, P = 0.90) was associated with EF in NCU patients.

## Discussion

Our systemic review and meta-analysis revealed that pneumonia, atelectasis, a >24-h MV, low GCS score, inability to follow commands (especially the command to close eyes), thick secretion, and no intact gag reflex predicted EF. Sex, secretion volume, coughing with suctioning, and the inability to follow one command among showing two fingers, wiggling the toes, and coughing on command did not predict EF. Some TWPs poorly predicted EF in NCU patients.

EF appeared to be particularly common in NCU patients with pneumonia and atelectasis, which reduce the vital capacity and tidal volume and severely interfere with extubation. Aggressive respiratory treatment should be implemented to prevent these complications and reduce the rate of EF.

Notably, our systematic review and meta-analysis found that some TWPs predicted EF poorly in the neurocritical population. This could be explained as follows. First, NCU patients are often intubated for airway protection caused by brain injury rather than lung pathology, which is originally measured by TWPs [6]. Therefore, in patients with primary neurological dysfunction, TWPs poorly predict EF. Second, TWPs were proved to fail in predicting other ICU populations as well deriving from low weaning predictor accuracy or the nature of failed weaning trials [1]. Finally, TWPs measure a patient's breathing function in a relatively rested state instead of assessing the forced maneuver of clearing the airway, which is imperative in judging whether a patient intubated solely for neurological reasons should be extubated [12].

The finding that TWPs failed to predict EF in neurocritical patients further supported the utility of neurologic abilities to predict extubation outcomes. Our study showed that the GCS score is an ideal predictor. This may be because patients with a lower level of consciousness have larger amounts of secretions and more severe cough or swallow deficiencies, all of which have been described by others as risk factors for EF [22]–[29]. However, some studies have found no association between the GCS score and EF [3], [26]. The degree of cranial nerve dysfunction and/or bulbar dysfunction is reportedly a likely contributor to the inability to protect the airways; this generally cannot be ascertained by the GCS score, although possible correlations with the GCS score have been found in some cases [13]. Nevertheless, most studies considered a GCS score of >7T to be a consideration for extubation [7], [9], [12], [13], [17], and patients for whom the consciousness was the dominant limitation to extubation success were thus excluded [7]. These inconsistent results require further investigation of the indications for extubation among patients with low GCS scores. According to the current study, however, a lower consciousness level as assessed by the GCS score (7–9T) was correlated with EF in NCU patients with a GCS score of ≧7.

The inability to follow commands and the inability to follow the command of closing the eyes in particular were associated with EF. The ability to follow commands represents, to some extent, the patient's mental status, which can affect the extubation outcome. Closing eyes on command, showing two fingers, wiggling the toes, and coughing on command require a relatively preserved consciousness, attention, intact long-tract motor pathways, and muscle coordination [7]. In particular, closing the eyes on command may represent arousal to a novel stimulus or a startle reaction rather than a volitional event [7]. In our study, however, the inability to follow the command of showing two fingers, wiggling the toes, and coughing on command did not predict EF. Further research should be undertaken to explore the explanation for this phenomenon.

The secretion texture (thick), but not the secretion volume, predicted EF in NCU patients. However, the qualitative estimates of sputum thickness and volume are subjective. Moreover, the lack of a quantitative standard limits their interpretation and generalizability [7]. As one aspect of airway protection, an intact gag reflex predicts EF well; however, Coplin [3] found that 89% of brain-injured patients with an absent or weak gag were successfully extubated. Coughing with suctioning was not associated with extubation outcomes in our review. Coplin [3] found that an adequate spontaneous cough was associated with successful extubation. This might be theoretically explained by the fact that spontaneous coughing indicates the capacity for active airway protection, while coughing with suctioning is only a passive reflex.

There are some limits in our review. First, because of deficiencies in the data, only the main predictors drawn from the articles were analyzed. Second, our study was subject to heterogeneity in some of the inclusion criteria, as well as relatively small sample sizes and numbers of articles that analyzed the predictors representing airway protection, which may have caused potential public bias. Finally, all articles included in our review were cohort studies. Additional randomized controlled trials are needed for a systematic review.

In conclusion, this systemic review and meta-analysis has shown that pneumonia, atelectasis, a >24-h MV, low GCS score (7–9T), inability to follow commands (especially the command to close the eyes), thick secretion, and no intact gag reflex are significant factors in predicting EF. On the other hand, sex; secretion volume; coughing with suctioning; the inability to follow one command among showing two fingers, wiggling the toes, and coughing on command; and some TWPs failed to show statistical significance. Clinicians should pay more attention to these factors when extubating neurocritical patients.

## Supporting Information

Checklist S1 **PRISMA 2009 Checklist.**
(DOC)
